# An adaptive approach to machine learning for compact particle accelerators

**DOI:** 10.1038/s41598-021-98785-0

**Published:** 2021-09-28

**Authors:** Alexander Scheinker, Frederick Cropp, Sergio Paiagua, Daniele Filippetto

**Affiliations:** 1 Applied Electrodynamics Group, Los Alamos National Laboratory, Los Alamos, NM 87545 USA; 2 Department of Physics and Astronomy, University of California Los Angeles, Los Angeles, CA 90095 USA; 3 Lawrence Berkeley National Laboratory, One Cyclotron Road, Berkeley, CA 94720 USA

**Keywords:** Information theory and computation, Applied mathematics, Computational science, Experimental particle physics

## Abstract

Machine learning (ML) tools are able to learn relationships between the inputs and outputs of large complex systems directly from data. However, for time-varying systems, the predictive capabilities of ML tools degrade if the systems are no longer accurately represented by the data with which the ML models were trained. For complex systems, re-training is only possible if the changes are slow relative to the rate at which large numbers of new input-output training data can be non-invasively recorded. In this work, we present an approach to deep learning for time-varying systems that does not require re-training, but uses instead an adaptive feedback in the architecture of deep convolutional neural networks (CNN). The feedback is based only on available system output measurements and is applied in the encoded low-dimensional dense layers of the encoder-decoder CNNs. First, we develop an inverse model of a complex accelerator system to map output beam measurements to input beam distributions, while both the accelerator components and the unknown input beam distribution vary rapidly with time. We then demonstrate our method on experimental measurements of the input and output beam distributions of the HiRES ultra-fast electron diffraction (UED) beam line at Lawrence Berkeley National Laboratory, and showcase its ability for automatic tracking of the time varying photocathode quantum efficiency map. Our method can be successfully used to aid both physics and ML-based surrogate online models to provide non-invasive beam diagnostics.

## Introduction

Machine learning (ML) methods, such as deep neural networks, can learn relationships between the components of and predict the outputs of complex physical systems such as phases of matter^1^ and extreme events in complex systems^2^. Consider an n-dimensional complex physical system whose evolution is described by a system of dynamic equations

1 $$\begin{aligned} \frac{\partial \mathbf {x}(\mathbf {p},t)}{\partial t} = \mathbf {F}(\mathbf {x}(\mathbf {p},t),\mathbf {p}), \end{aligned}$$

where $\mathbf {x} = (x_1,\dots ,x_n)$ is a vector of physical quantities within the system such as atomic positions or energies, $\mathbf {p}=(p_1,\dots ,p_m)$ is a vector of controlled system parameters such as voltages or chemical concentrations, and $\mathbf {F}$ represents the nonlinear dynamics governing the physical system which may include partial derivatives with respect to the components of both $\mathbf {x}$ and $\mathbf {p}$. Associated with a system such as (1) there is usually some physical-meaningful measurement, $M\left( \mathbf {x}(\mathbf {p},t),\mathbf {p}\right)$, which depends on the parameter settings $\mathbf {p}$ and on the entire resulting time-history of $\mathbf {x}(\mathbf {p},t)$. For example, such a measurement may be material properties such as strength of a complex alloy or the results of a high energy physics experiment.

There are cases where the system (1) is so large and complex that an accurate analytical representation of $\mathbf {F}$ is unknown. There are also cases in which an analytic physics-based model of $\mathbf {F}$ exists, but is so computationally expensive that it cannot be numerically simulated over large length and time scales and requires lengthy computations with high performance computing resources. In either of those two cases, ML methods can be useful for learning a representation of the system that quickly maps parameter settings to measurements. Tool such as recurrent neural networks can handle entire time-series trajectories and reinforcement learning approaches can model analytically unknown reward functions and their corresponding optimal controllers for analytically unknown systems. Such ML-based approaches can learn these representations directly from raw data, whether the data comes from measurements or simulations, by using large collections of input-output pairs,

2 $$\begin{aligned} D = \left\{ \left( M_i, \mathbf {p}_i \right) \right\} _{i=1,\dots ,N}, \end{aligned}$$

to learn parameter to measurement maps

3 $$\begin{aligned} \mathbf {p}\Longrightarrow {\hat{M}}\left( \mathbf {w},\mathbf {b}, \mathbf {p} \right) \approx M\left( \mathbf {x}(\mathbf {p},t)\right) , \end{aligned}$$

where $\mathbf {w}$, $\mathbf {b}$ are the weights and biases of the ML model whose values are found by minimizing the error between measurements and predictions based on data set *D* as in (2). Once such maps are learned they can be used for applications such as fast searches over large parameter spaces, to extract correlations and physics from experimental data, and to guide the design of new systems.

To give a few concrete examples, various ML methods have now been demonstrated for a wide range of systems such as molecular and materials science studies^3^, for use in optical communications and photonics^4^, to accurately predict battery life^5^, to accelerate lattice Monte Carlo simulations using neural networks^6^, for studying complex networks^7^, for characterizing surface microstructure of complex materials^8^, for chemical discovery^9^, for noninvasive identification of Hypotension using convolutional-deconvolutional networks^10^, for active matter analysis by using deep neural networks to track objects^11^, for imputation of missing physiological waveform data by using convolutional autoencoders^12^, for optimizing operational problems in hospitals^13^, for cardiovascular disease risk prediction^14^, for particle physics^15^, for antimicrobial studies^16^, for pattern recognition for optical microscopy images of metallurgical microstructures^17^, for learning Perovskit bandgaps^18^, for real-time mapping of electron backscatter diffraction (EBSD) patterns to crystal orientations^19^, for speeding up simulation-based accelerator optimization studies^20^, for Bayesian optimization of free electron lasers (FEL)^21^, for temporal power reconstruction of FELs^22^, for various applications at the Large Hadron Collider (LHC) at CERN including optics corrections and detecting faulty beam position monitors^23–26^, for reconstruction of a storage ring’s linear optics based on Bayesian inference^27^, to analyze beam position monitor placement in accelerators to find arrangements with the lowest probable predictive errors based on Bayesian Gaussian regression^28^, for temporal shaping of electron bunches in particle accelerators^29^, for stabilization of source properties in synchrotron light sources^30^, and to represent many-body interactions with restricted-Boltzmann-machine neural networks^31^.

One challenge faced in applying ML methods to complex physical systems is if the systems have hidden time-varying characteristics which describe the parameter-to-physical system relationships. A simple example of a time-varying system is

4 $$\begin{aligned} {\dot{x}} = x + p_h(t)p, \end{aligned}$$

where $p_h(t)$ is an unknown time-varying parameter and *p* is an input parameter. By hidden parameters we mean parameters whose settings are not directly observable. Note that in this simple example (4) if there is no feedback ($p\equiv 0$) the system is unstable and exponentially diverges. If the unknown time-varying function $p_h(t)$ repeatedly changes sign, such as

5 $$\begin{aligned} p_h(t) = \cos (\omega (t) t), \end{aligned}$$

where the frequency of oscillation is itself also an uncertain time-varying system, it is a major challenge for data-based methods because the input *p* to output *x*(*t*) relationship changes repeatedly in an unpredictable way, resulting in the smallest error for a large data set being an average approximation of $p_h(t)\equiv 0$. Such systems are also challenging for standard feedback controls such as proportional integral derivative (PID) feedback which must assume a known sign of $p_h(t)$ and fails spectacularly, destabilizing the system even more whenever $p_h(t)$ changes sign. Although many model-independent adaptive feedback control methods exist^32^, the problem of designing a stabilizing feedback controller for unknown time-varying systems with unknown control directions such as (4) with analytical proof of convergence was not accomplished until 2013 by utilizing nonlinear time-varying adaptive feedback and Lyapunov stability analysis methods^33^. Another form of hidden time-varying characteristics is when the initial conditions of a system are themselves time-varying, not easily measurable, and influence the dynamics of the system in a non-trivial way.

In both of the above cases, if either the dynamics-to-parameters relationships or the initial conditions vary with time, then the accuracy of data-based predictions will start to degrade as the system changes to such an extent that it is no longer accurately represented by the data that was originally used to train the ML model. Although powerful ML methods such as domain transfer and re-training are available, for large quickly changing systems with many parameters they may become infeasible. Furthermore, for many systems even if it was possible to quickly collect new data to re-train an ML model for new conditions, sometimes such measurements are highly invasive and cannot be accomplished in real time without interrupting regular operations. Including time as an extra input to the ML tool and attempting to learn the time dependence of the system from data is only possible for simple cases in which the changes are predictable, such as monotonic or periodic changes. Although ML methods for time-series data exist, such as LSTM-based neural networks, those also need to be trained with consistent data and if the dynamics that govern that data change then the predicted time-series will be wrong. Model-independent adaptive feedback methods which respond in real time to un-modeled disturbances and changes face their own major limitations. Although adaptive methods are able to handle time-varying systems they are usually local in nature and can require lengthy tuning or get stuck in local minima especially for large complex systems with many parameters.

**Figure 1:**
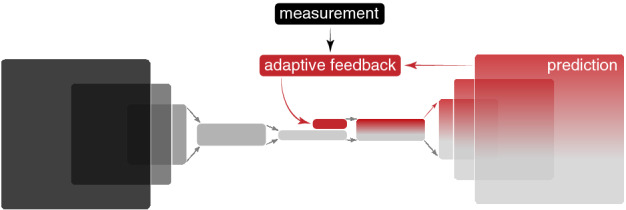
A high level overview of the adaptive ML approach for time-varying systems with flexibility for adaptation added by including an input vector of adaptively tuned parameter that is concatenated together with a vector output of a dense layer within a convolutional neural network after the encoder and before the generative section.

In this paper, we present an approach to deep learning for time-varying systems which combines the ability of data-based ML methods to learn global complex relationships directly from data with the robustness of model-independent adaptive feedback to handle unknown time-varying conditions. In particular we focus on encoder-decoder type convolutional neural networks (CNN) which encode high dimensional inputs to a low dimensional latent space of dense layers. Our approach adds flexibility into the generative half of the CNN by adding adaptively-guided feedbacks into the low-dimensional dense layers of the network. A high level overview of this approach is shown in Fig. 1. Our adaptive ML method is designed for time-varying systems of the form

6 $$\begin{aligned} \dot{\mathbf {x}}(\mathbf {p},\mathbf {p}_h(t),t) = \mathbf {F}(\mathbf {x}(\mathbf {p},\mathbf {p}_h,t),\mathbf {p},\mathbf {p}_h(t),t), \end{aligned}$$

where as before $\mathbf {x}=(x_1,\dots ,x_n)$ are physical quantities whose measurements are of interest, $\mathbf {p}=(p_1,\dots ,p_m)$ are adjustable parameters, and the new term $\mathbf {p}_h(t) = \left( p_{h,1}(t),\dots ,p_{h,m_h}(t) \right)$ are hidden time-varying parameters that influence the time-varying system dynamics $\mathbf {F}$, but are not directly observable. Our goal is to develop an adaptive ML-based model ${\hat{M}}$ for predicting a time-varying measurement *M* of states of the system (6). Our model is of the form

7 $$\begin{aligned} {\hat{M}}\left( \mathbf {w},\mathbf {b},\mathbf {p},\hat{\mathbf {p}}_h(t) \right) \approx M\left( \mathbf {p},\mathbf {p}_h(t), t \right) , \quad \dot{\hat{\mathbf {p}}}_h(t) = \mathbf {f}_h(\hat{\mathbf {p}}_h,t,C(t)), \quad C(t) = \mu \left( {\hat{M}}(t) \right) , \end{aligned}$$

where as before the $\mathbf {w}$, $\mathbf {b}$ are network weights and biases that are trained from data sets, $\mathbf {p}$ are known set parameters, and the $\hat{\mathbf {p}}_h(t)$ are time-varying tunable parameters that are adjusted dynamically according to an adaptive algorithm whose dynamics are represented as $\mathbf {f}_h$. Although we write $\hat{\mathbf {p}}_h(t)$ to represent the fact that these adjustable parameters are supposed to track the unknown hidden parameters $\mathbf {p}_h(t)$, they can also include more abstract quantities such as additional neural network inputs, weights, or biases. The adjustable parameters are adaptively tuned based on feedback that depends on some function of the prediction, $C(t) = \mu \left( {\hat{M}}(t) \right)$, where we are only showing the time arguments of the functions to emphasize that they may be drifting and changing. For example, for the application described below, our ML model’s goal is to predict the time-varying input beam distribution $I_i(t)$ of an accelerator based on the measurement of an output beam distribution $I_o(t)$ at another location further down the beam line. This is done because the input beam distribution is not easily directly measurable and therefore our adaptive tuning adjusts both the neural network’s estimate of $I_i(t)$ as well as a hidden parameter which represents a time-varying magnet in the accelerator lattice. The adaptive feedback is performed based on a comparison between the measured accelerator output beam $I_o(t)$ and that of an online model whose input is the ML-based approximated distribution and whose time-varying magnet setting is also adaptively adjusted. This allows us to make adjustments in real time purely based on available diagnostics without having to re-train the ML model.

Our approach of combining external feedbacks with neural networks is analogous to a natural phenomenon in biological systems in which networks of neurons interact with each other and are controlled by external feedback loops and other networks^34^. For example recent studies have shown that the challenging task of synchronization between neuron networks can be achieved by feedback controls in the presence of signal delay, noise, and external disturbances^35,36^, and that resonance can be excited and controlled in complex neuron systems by using external feedback signals including chaotic resonances^37,38^. Some initial simplified adaptive ML studies have also begun on coupling the outputs of CNNs to adaptive feedback for real-time accelerator phase space control^39^ and for predicting 3D electron density distributions for 3D coherent diffraction imaging^40^.

**Figure 2:**
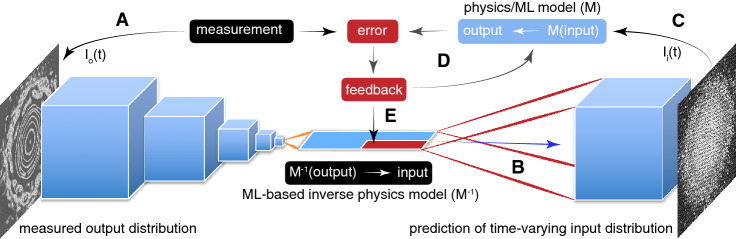
Overview of an adaptive ML approach for ML-based time-varying inverse models. In our application a convolutional neural network is trained to represent an inverse physics model $M^{-1}$ which maps an output distribution $I_o(t)$ to its associated input distribution $I_i(t)$ for a complex system (**A**,**B**). The ML-based estimate of the input $I_i$ is then fed into a physics or ML-based surrogate model (**C**) and the model’s output is compared to measurements to quantify an error which is used to guide iterative feedback (**D**). In this adaptive approach the dense layers of the network include inputs from an adaptive feedback loop which are perturbed based on model or measurement-based errors (**E**).

In the remainder of this paper we focus on ML-based inverse physics models for time-varying systems, an overview of the approach is shown in Fig. 2, which is a specialized form of the general setup in Fig. 1. This approach is motivated by the fact that very sophisticated and accurate physics models as well as ML-based surrogate models have been developed for many complex systems, which could benefit from accurate estimates of the time-varying initial distributions which are used as their inputs. In particular we will demonstrate our method for particle accelerators and their charged particle beams for which our inverse modeling approach is motivated by sophisticated beam dynamics models which have the potential to serve as non-invasive beam diagnostics if their parameters and input beam distributions could be matched to actual accelerators.

Particle accelerators are powerful tools for scientific research such as imaging of nuclear motion by ultrafast electron diffraction (UED)^42,43^ reaching femtosecond temporal resolution^44^ allowing for the visualization of lattice dynamics^45^ and monitoring the spatiotemporal dynamics of excited atoms or molecules^46^, imaging dynamic phenomenon at free electron lasers (FEL)^47^, and generating GV/m accelerating fields in plasma wakefield accelerators (PWFA)^48^. Major challenges faced by accelerators are the ability to quickly and automatically control the phase space distributions of accelerated beams such as custom current profiles, and switching between different experiments with order of magnitude changes in beam properties, which can require hours of hands on tuning due to limited non-invasive diagnostics, time variation of accelerator parameters, and complex time varying beams.

**Figure 3:**
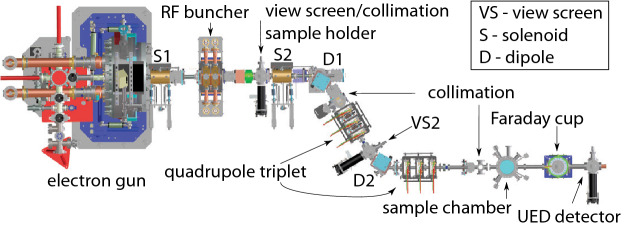
Overview of the HiRES UED adapted from^41^.

Intense bunches of $10^5{-}10^{10}$ particles are complex objects whose 6D phase space $(x,y,z,p_x,p_y,p_z)$ dynamics are coupled through collective effects such as space charge forces and coherent synchrotron radiation^49^. Accelerators generate extremely short bunches: 30 fs bunches at the SwissFEL X-ray FEL^50^, sub 100 fs bunches for UEDs^51^, and picosecond bunch trains for UEDs and multicolor XFELs^52^. The High Repetition-rate Electron Scattering apparatus (HiRES, shown in Fig. 3) at Lawrence Berkeley National Laboratory (LBNL), accelerates pC-class, sub-picosecond long electron bunches up to one million times a second (MHz), providing some of the most dense 6D phase space among accelerators at unique repetition rates, making it an ideal test bed for advanced algorithm development^41,53^.

Advanced diagnostics are important for particle accelerator applications because the dynamics of intense charged particle beams are dominated by complex nonlinear collective effects such as space charge forces and coherent synchrotron radiation in a 6 dimensional (6D) phase space $(x,y,z,p_x,p_y,p_z)$. Non-invasive real-time phase space diagnostics would be of great benefit to all particle accelerators as they would provide information which could guide adaptive feedback mechanisms. Automatic fast feedback could then be used for real-time beam tuning, such as quickly changing between various experiments at free electron lasers by modifying the energy vs time phase space of the beam, for creating custom tailored current profiles of intense bunches in plasma wakefield acceleration experiments, and for controlling the transverse bunch shapes for ultra fast electron diffraction (UED) microscopy beam lines.

Although physics and machine learning-based surrogate models can potentially serve as non-invasive beam diagnostics, the main challenges they face are uncertain and drifting accelerator parameters and uncertain initial beam distributions at the entrance of an accelerator to be used for initial conditions. Such distributions drift with time requiring lengthy measurements that interrupt accelerator operations. For example, it has been demonstrated that an online model can be adaptively tuned in order to provide a virtual diagnostic for the time-varying longitudinal phase space (LPS) of an electron beam at the Facility for Advanced Accelerator Experimental Tests (FACET) at SLAC National Accelerator Laboratory by comparing the model’s predictions to a non-invasive energy spread spectrum measurement^54^. Although the adaptive model tuning approach at FACET was able to track the beam’s LPS in real time its accuracy was limited by the fact that there was no access to a good estimate of the time varying input beam distribution. Another set of virtual LPS diagnostics at FACET and at the LCLS free electron laser (FEL) were then developed which utilized neural networks to instantly map accelerator parameters to LPS measurements^55^. A third approach to virtual LPS diagnostics was recently developed at the LCLS which used neural networks with spectral inputs as well as parameter settings resulting in higher accuracy predictions^56^. Recently encoder-decoder CNNs have also been demonstrated with measured beam data at the European XFEL to provide extremely high accuracy (768 × 1064 pixel images) predictions of the beam’s LPS and have also demonstrated an innovative method in which once the decoder half is trained and fixed, multiple different encoders can be used for various working points without having to re-train the decoder^57^. Simulation-based surrogate modeling studies have also been performed for mapping LPS distributions between various locations in accelerators^58^. All such ML methods could also greatly benefit from adding an estimate of the time-varying initial beam distribution as an input via the approach proposed in this paper or from adding adaptive tuning to some of their dense layers to make them more robust to time-varying initial beam distributions.

## Methods

In the present work we utilized an adaptive CNN-based inverse model for mapping output beam measurements measurements directly to the learned principal components that represent beam inputs at the High Repetition-rate Electron Scattering apparatus (HiRES, shown in Fig. 3) beamline at Lawrence Berkeley National Laboratory (LBNL), which accelerates pC-class, sub-picosecond long electron bunches up to one million times a second (MHz), providing some of the most dense 6D phase space among accelerators at unique repetition rates, making it an ideal test bed for advanced algorithm development^41,53^.

The first step was to collect data to learn a basis with which to represent a distribution of interest. We were interested in representing the time-varying input beam distribution $I_i(t)(x,y)$ at the photocathode of the electron gun of HiRES. We started by collecting laser spot images on the HiRES photocathode over several days. The transverse electron beam density distribution $\rho (x,y)$ created by a laser pulse of intensity *I*(*x*, *y*) is given by $\rho (x,y) = I(x,y)\times QE(x,y)$. For our experiments we used a laser spot of small transverse size of roughly 200 × 200 μm^2^, an area small enough so that no significant quantum efficiency variations were expected and we could relate laser intensity directly to an initial electron bunch charge distribution.

In order to increase the generality of our approach the laser images were then each rotated through 360° at steps of 1° generating a total of 1800 52 × 52 pixel images. These images were then stretched out as 1 × 52^2^ dimensional vectors and stacked in a 2704 × 1800 data matrix *D*. Considering this as a collection of 1800 vectors from a 2705 dimensional space, we then carried out a principal component analysis (PCA)^59^, a method closely related to singular value decomposition for finding lower dimensional representations of higher dimensional spaces, such as the method for finding the most important components of a large complex beam line^60^. PCA was performed by subtracting the mean of each column of *D* and then factorizing the data matrix *D* via singular value decomposition to calculate the score matrix *T* according to

8 $$\begin{aligned} D = U\Sigma W^T, \quad T = DW = U\Sigma W^TW = U\Sigma , \end{aligned}$$

whose components can be used in linear combinations as a basis with which to represent the original images.

We then generated input beam distributions $I_i$ as linear combinations of $N_{pca}$ PCA components as

9 $$\begin{aligned} I_{i,N_{pca}} = \sum _{n=1}^{N_{pca}}\alpha _{i,n} \times \mathrm {PC}_n. \end{aligned}$$

**Figure 4:**
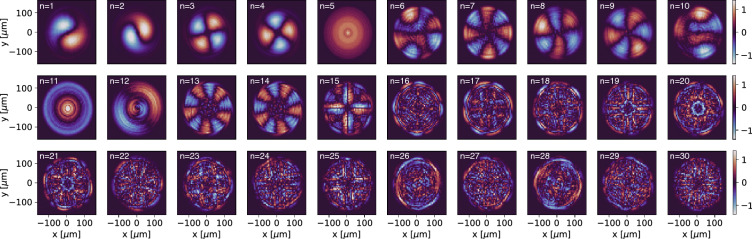
The first 30 principal components (normalized) of the input electron beam distribution are shown. The most dominant modes are $n=1$ to $n=15$, after which the components are seen to represent very fine details.

Although hundreds of PCA components were required to represent $>95\%$ of the variance in our data, we wanted to significantly reduce the dimensionality of our problem. The first 30 principal components $(\mathrm {PC}_1,\dots ,\mathrm {PC}_{30})$ are shown in Fig. 4 and qualitatively the most dominant modes are the first 15. To quantify the accuracy of reconstruction as a function of the number of PCA components used, we defined the error

10 $$\begin{aligned} \mathrm {error} = 100 \times \sum _{x,y}\left| I_i - I_{i,N_{pca}} \right| \bigg / \sum _{x,y}I_i . \end{aligned}$$

We then represented the 5 measured input beam distributions according to (9) and found that the error quickly dropped and leveled off at $\sim$ 8% for $N_{pca}=15$ after which it decayed very slowly so we used the first 15 images shown in Fig. 4 as the basis vectors for representing input beam distributions for HiRES. This limits the lowest error we can achieve for real distributions at 8%, but greatly speeds up real time adaptive tuning.

Next we calibrated a General Particle Tracer (GPT) model using a generative neural network surrogate model (SM) to quickly map parameter settings to output beam distributions^61,62^ by a high dimensional parameter search (including average beam energy $(x_p,y_p,z,E)$) to match predictions to measured beam data. The calibrated GPT model was then used to generate 51 thousand pairs $(X_i,\mathbf {y}_i)$, with each $\mathbf {y}_i = (\alpha _{i,1},\dots ,\alpha _{i,15})$ representing 15 PCA coefficients sampled from a normal distribution to generate a random input beam distribution ${\hat{I}}_{i}$ as in (9), and $X_i$ being the 51 × 51 image of the (*x*, *y*) output beam distribution at the first view screen of HiRES, generated by the GPT model using ${\hat{I}}_{i}$ as input.

**Figure 5:**
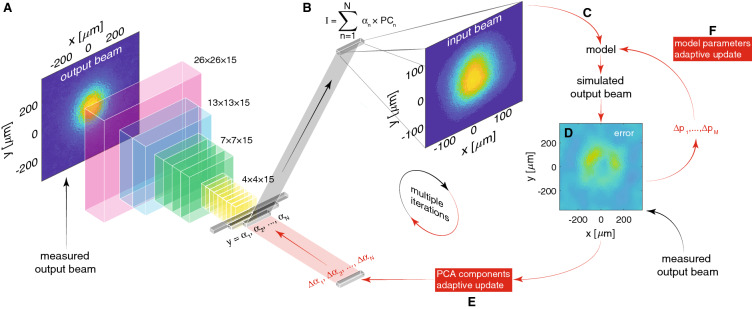
(**A**) The measured beam output is fed into the CNN of convolutional layers with LeakyReLU activation functions followed by dense layers with ReLU activations. The CNN outputs principal component coefficients $(\alpha _1,\dots ,\alpha _N)$ which generate an input beam distribution (**B**) used as an input to the simulation (**C**). (**D**) Simulation output is compared to the measured beam output to adaptively fine tune both the principal components (**E**) and model parameters (**F**).

We then trained a CNN to map output beam distributions to the accelerator’s input beam distribution as shown in Fig. 5A. For the generative half of the CNN, instead of allowing the CNN to build in an arbitrary latent space by a standard approach such as transposed convolution layers, our approach guided the CNN to produce physically significant quantities which were the PCA components representing the basis out of which to build our input beam distributions.

**Figure 6:**
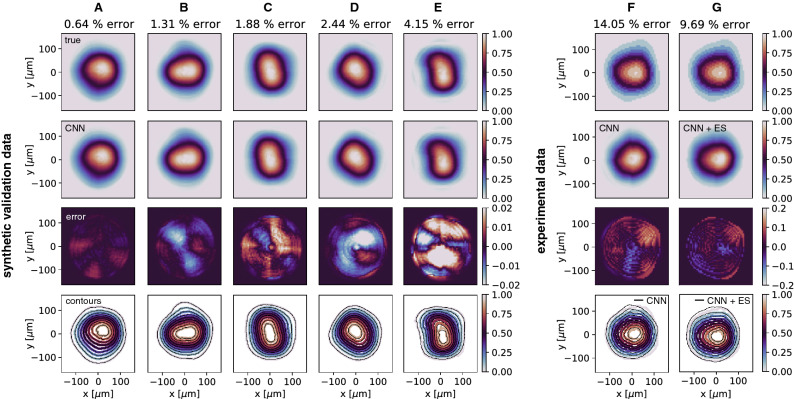
(**A**–**E**) Comparing input distributions to the CNN predictions reconstructed from the predicted PCA component coefficients. 5 examples were chosen from the 1000 test distributions and show the CNN’s best prediction (**A**), worst prediction (**E**), and a uniformly spaced range between (**B**–**D**). (**F**) The CNN’s prediction is shown for an experimentally measured beam output and compared to the experimentally measured beam input. (**G**) Results of the prediction from (**F**) fine tuned via ES.

The CNN was tested on 1000 unseen input-output pairs and had an average error as defined in (10) of 1.88% with a standard deviation of 0.56%. Next, we used an experimentally measured output beam distribution as the input to the CNN and were able to predict the corresponding measured input beam distribution with an accuracy of 14.05%. Finally, the CNN’s prediction was fine tuned using an iterative adaptive feedback approach with each new input distribution fed into GPT giving a new output distribution, as shown in Fig. 5B–E, resulting in prediction accuracy of 9.69%. A detailed view of adaptive CNN predictions is shown in Fig. 6.

The input beam distributions generated by the CNN were then fed into the GPT model to produce output beam estimates, which were compared to measurements. The error between predicted and measured beam outputs was used to drive an adaptive feedback loop which was connected back into the dense layer of the CNN to adaptively tune the CNN’s generative layer’s predictions. The adaptive method used was extremum seeking (ES) for high dimensional dynamic systems of the form

11 $$\begin{aligned} \dot{\mathbf {x}} = \mathbf {f}(\mathbf {x}(\mathbf {p},t),\mathbf {p},t), \quad {\hat{y}} = y(\mathbf {x}(\mathbf {p},t),\mathbf {p},t) + n(t), \end{aligned}$$

where $\mathbf {f}$ is an analytically unknown time-varying nonlinear function, $\mathbf {x}$ are measurable system values, $\mathbf {p}$ are adjustable parameters, and ${\hat{y}}$ is a noisy measurement of an analytically unknown function *y* that can be maximized by adjusting $\mathbf {p}$ according to

12 $$\begin{aligned} \frac{dp_j}{dt} = \sqrt{2\alpha \omega _j} \cos (\omega _j t + k {\hat{y}}), \end{aligned}$$

where $\omega _j \ne \omega _i$ for $i \ne j$. The term $\alpha > 0$ is the dithering amplitude and can be increased to escape local minima and $k>0$ is feedback gain. For large $\omega _i$, the dynamics of (12) are, on average $d\mathbf {p}/dt = -k\alpha \nabla _{\mathbf {p}}y$, a gradient descent (for $k>0$) or ascent (for $k<0$), with respect to $\mathbf {p}$, of the actual, analytically unknown function *y* although feedback is based only on the noisy measurements ${\hat{y}}(t)$, for details and proofs see^33,63,64^.

## Results

### Demonstration at HiRES UED

In our approach the dynamic system of interest was the HiRES beamline and its GPT-based simulation. Our inverse model was designed to map HiRES output beam distributions to their associated input distributions and the CNN parameters being tuned, $\mathbf {p}$, were dense layers which created the PCA coefficients defining the input beam distribution, as well as a hidden parameter within the HiRES simulation represented by the solenoid magnet setting.

Our adaptive feedback was guided by maximizing the structural similarity index (SSIM) between a measured output beam distribution $I_o(t) = \rho _m(x,y,t)$, and the GPT simulation-based output beam distribution ${\hat{I}}_0(t) = \rho _s(x,y,t)$, both of which were smoothed with a Gaussian filter of variance 2 and normalized to a range of [0, 1]. SSIM is defined as

13 $$\begin{aligned} \mathrm {SSIM}(\rho _m,\rho _s) = \frac{\left( 2\mu _m\mu _s + c_1\right) \left( 2\sigma _{ms} + c_2 \right) }{\left( \mu _m^2+\mu _s^2+c_1 \right) \left( \sigma _m^2+\sigma _s^2+c_2 \right) }, \end{aligned}$$

where $\mu _m$ and $\mu _s$ are mean values of the measured and simulated distributions, $\sigma _m^2$ and $\sigma _s^2$ are their variance, $\sigma _{ms}$ is the covariance, and $c_1 = c_2 = 10^{-4}$^65^. The SSIM value lies within the range $[-1.0,1.0]$ with a value of 1.0 for images that are exactly the same.

To demonstrate the robust adaptive capability of our approach to simultaneously track both changing accelerator parameters and time-varying initial beam distributions, we performed a study running three sets of GPT simulations. In the first simulation, representing HiRES, an input beam was defined and its PCA components as well as the current of solenoid S1 were changed over time, resulting in a large variation of both the input and output beam. Based on that output beam of the first simulation, the CNN alone was used to predict the PCA components which generated an input to a second simulation to predict the output beam. This second simulation quickly diverged from the first because the system changed from the one that had been used to train the CNN. For a third simulation, the adaptive feedback algorithm (12) was also applied with *y* as in (13), which adaptively tuned both the PCA components and the current of solenoid S1 in order to track the time-varying output distribution of the first simulation, as shown in Fig. 5B–F.

**Figure 7:**
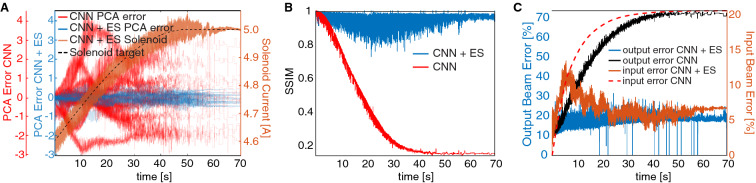
(**A**) PCA component tracking error shown with CNN alone and with CNN $+$ ES, in the latter case the solenoid strength is also tracked. (**B**) CNN $+$ ES tracks the output beam. (**C**) Output and input beam distribution errors continuously minimized.

**Figure 8:**
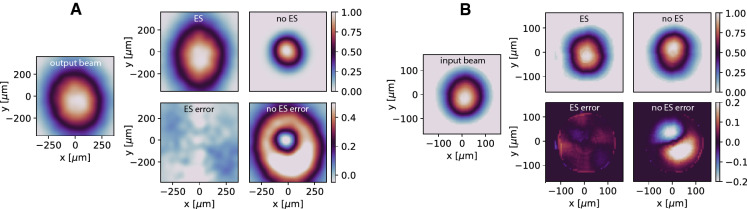
(**A**) The output beam distribution prediction adaptively tracked by tuning solenoid current and PCA components. (**B**) Input beam distribution is predicted with high accuracy.

**Figure 9:**
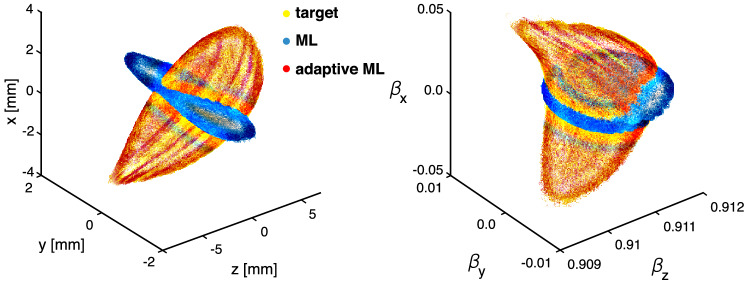
6D phase space predictions with and without adaptive ML.

Figure 7A shows the error of the PCA component predictions and demonstrates that the CNN + ES setup was able to simultaneously track both the time-varying solenoid current and the PCA components. Figure 7B compares the SSIM of the output beams with and without ES for tracking, (C) shows the percent error (10), and Fig. 8 compares the target output beam and input beam with and without using ES. Using the adaptively tracked input distribution and S1 setting we simulated a 1.6 pC bunch with 3D space charge and compared the 6D phase space to that which would have been generated by the exact correct values as well as to the CNN alone in Fig. 9, showing an almost exact match of the 6D phase space.

Note that we chose solenoid S1 as the hidden time-varying parameter to be tracked only for the purpose of demonstrating this adaptive technique. In general many such parameters can be adaptively tuned simultaneously such as multiple quadrupole magnets which suffer from hysteresis.

**Figure 10:**
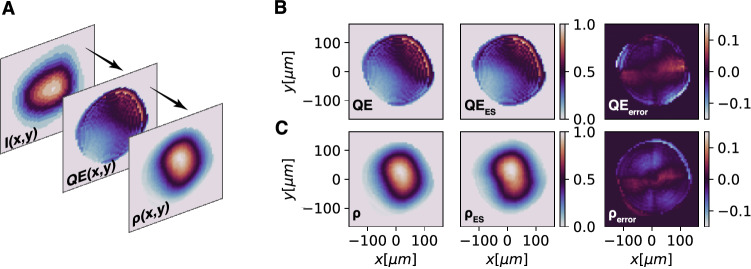
(**A**) Schematic of laser image, quantum efficiency, and the resulting beam density. (**B**,**C**) Target QE and beam density compared to adaptive reconstructions.

Finally, to demonstrate the ability of the approach in Fig. 7 to track time-varying beamline paramters, we generated an artificial quantum efficiency map *QE*(*x*, *y*), and used the measured laser intensity *I*(*x*, *y*) to construct a beam distribution $\rho = I\times QE$ which was used as input to the GPT model. The output was fed into the CNN whose initial guess of $\rho$ was then adaptively fine tuned. The QE of the cathode was then reconstructed as $QE(x,y) = \rho (x,y)/I(x,y)$, as shown in Fig. 10.

## Discussion

We have proposed an approach for adaptively tuning the parameters of both an online physics model and encoder-decoder CNN-based inverse physics model by using an extremum seeking model-independent feedback method. This approach results in an overall adaptive ML setup which automatically compensates for unknown time-varying changes to accelerator components (such as magnets, and to unknown changes in the accelerator’s input beam distribution), by tuning inputs to the low dimensional dense layers preceding the decoder section of the CNN. We showed how the precision of the prediction is greatly improved when an adaptive component is added to the CNN, resulting in a much improved robustness of the model and broader application space. Adjustments to the input values were only based on outputs and online non-invasive system measurements. Although demonstrated here for a particle accelerator, such method can be applied to a wide range of complex time-varying systems with limited diagnostics.

We have demonstrated our method for adaptively tracking the time varying input beam distribution of the HiRES UED particle accelerator and the associated quantum efficiency (QE) when a measurement of the laser transverse profile at the cathode is available, for example through implementation of virtual cathode diagnostic. Our general approach can be applied at various particle accelerators to enable physics models to accurately map distributions between various locations to enable adaptive tuning. For example transverse deflecting radio frequency resonant cavity (TCAV)-based LPS measurements can be used to reconstruct the initial (*z*, *E*) LPS. Such model-based non-invasive diagnostics are possible because the rich physics in models are strong constraints on the dynamic maps between accelerator distributions. Given sufficiently rich measurements we are very unlikely to achieve a close match at one section of an accelerator without uniquely reproducing the correct input distribution.

Tracking non-stationary systems requires adaptive algorithms to be able to map measured outputs to unknown time-varying input(s). Isolating the correct changing input(s), together with its magnitude and time-scale, is an extremely complex problem, including multi-parameter optimization of the inverse physics problem. While this could be achieved using particle tracking codes (such as GPT), it would be incredibly computationally expensive and time consuming, and would practically prevent the method to be used in beamlines for real-time feedbacks. Powerful ML tools, such as convolutional neural networks, speed up operations by order of magnitudes (a single prediction as shown in Fig. 6F takes less than a millisecond), and can therefore efficiently be used for fine-tuned by the adaptive feedback and tracked over time, as shown in Figs. 6G and 7. We demonstrated that our approach is simultaneously tracking both the unknown input beam distribution and time-varying accelerator parameters.

Lastly, it is worth pointing out the limitations to the re-training free approach presented here. First, if the magnitude of the input beam distribution change it is such that the new input no longer falls within the span of the PCA-based basis vectors which we are using, the error between the real and predicted input will diverge. Second, if something in the system changes that is not part of the model or is not being adaptively tuned in the model, such as adding or removing a magnet from the beamline, then a new online model and a new experimental data set must be collected for this fundamentally different system.

In future work we plan to expand on our results by using several diagnostics simultaneously, including TCAV based LPS measurements and magnet tuning-based transverse phase space measurements. Our method can also be extended to 3D distributions $\rho (\mathbf {r})$ using 3D PCA components, spherical harmonics defining star-convex distribution boundaries $\partial \rho (\theta ,\varphi )$ as

14 $$\begin{aligned} \partial \rho (\theta ,\varphi ) = \sum _{l,m}\alpha ^m_lY^m_l(\theta ,\varphi ), \end{aligned}$$

where a convolutional neural network can be used to generate the spherical harmonics coefficients $\alpha _l^m$ which are then adaptively tuned. A more general approach is to generate distributions from combinations of radial basis functions (RBF) defined as

15 $$\begin{aligned} \rho (\mathbf {r}) = \sum _{n}\alpha _{n}e^{-\left( \mathbf {r}-\mathbf {r}_{c,n}\right) ^2/\sigma ^2_n}, \end{aligned}$$

where the neural network predicts the RBF amplitudes $\alpha _n$, centers $\mathbf {r}_{c,n}=(x_{c,n},y_{c,n},z_{c,n})$, and decay rates $\sigma _n$, all of which are adaptively tuned.
